# Retraction Note: BRD7 inhibits the Warburg effect and tumor progression through inactivation of HIF1α/LDHA axis in breast cancer

**DOI:** 10.1038/s41419-019-1864-y

**Published:** 2019-09-03

**Authors:** Weihong Niu, Yanwei Luo, Xinye Wang, Yao Zhou, Hui Li, Heran Wang, Yaojie Fu, Shanshan Liu, Shanghelin Yin, Jianglei Li, Ran Zhao, Yukun Liu, Songqing Fan, Zheng Li, Wei Xiong, Xiaoling Li, Guiyuan Li, Caiping Ren, Ming Tan, Ming Zhou

**Affiliations:** 1 The Affiliated Tumor Hospital of Xiangya Medical School, Central South University, Changsha, Hunan 410013 People’s Republic of China; 2 The Key Laboratory of Carcinogenesis of the Chinese Ministry of Health, The Key Laboratory of Carcinogenesis and Cancer Invasion of the Chinese Ministry of Education, Xiangya Hospital, Central South University, Changsha, Hunan 410078 People’s Republic of China; 3 Cancer Research Institute, Central South University, Changsha, Hunan 410078 People’s Republic of China; 4 The Second Xiang-Ya Hospital, Central South University, Changsha, Hunan 410011 People’s Republic of China; 5 Medical school of Xiangya, Central South University, Changsha, Hunan 410013 People’s Republic of China; 6 High Resolution Mass Spectrometry Laboratory of Advanced Research Center, Central South University, Changsha, Hunan 410013 People’s Republic of China; 7 Mitchell Cancer Institute, University of South Alabama, Mobile, AL 36604 USA

**Retraction to:** **Cell Death & Disease**

10.1038/s41419-018-0536-7 Published online 3 May 2018

The authors are retracting this article because after publication they became aware that there are errors in a number of the figures:

- In Fig. 2e the flow cytometry dot plots in the BRD7 groups for MCF-7 and MDA-MB-231 are similar (especially in Q1 and Q4).
- In Fig. 2e the Vector group is the same as the BRD7 plus LDHA group in MDA-MB-231 in Fig. 6d.
- In Fig. 2c the colony dish in the Vector group for MDA-MB-231 is the same as the BRD7 + LDHA group for MDA-MB-231 in Fig. 6b.
- In Fig. 5b the western blot (FLAG) is the same as that in Fig. 5g (BRD7).
- In Fig. 7b the loading control (β-tubulin) of MCF-7 is the same as CDK4 of MDA-MB-231 in Fig.7a.

All authors agree with this retraction.

